# Predictive Value of Normalized Lactate Load for Patients with Acute Type A Aortic Dissection: Based on the MIMIC-IV Database

**DOI:** 10.5761/atcs.oa.25-00092

**Published:** 2025-11-21

**Authors:** Qian Zhang, Jia Jiang

**Affiliations:** 1 The Second Department of Respiratory and Critical Care Medicine, Shaanxi Provincial People’s Hospital, Xi’an, Shaanxi, China; 2 Nephrology Department, Shaanxi Provincial People’s Hospital, Xi’an, Shaanxi, China

**Keywords:** normalized lactate load, aortic dissection, 30-day mortality, prognostic biomarker, Medical Information Mart for Intensive Care-IV

## Abstract

**Purpose:**

The objective of this study was to examine the correlation between normalized lactate load (NLL) and the 30-day mortality rate in patients with acute type A aortic dissection (AAAD) patients, as well as its predictive value for prognosis.

**Methods:**

Data were obtained from the Medical Information Mart for Intensive Care-IV database. The Cox model and restricted cubic spline (RCS) were used to assess the relationship between NLL and 30-day mortality in AAAD patients. Receiver-operating characteristic curves were plotted to evaluate the predictive value of NLL for 7-, 14-, and 30-day mortality. Kaplan–Meier (K–M) curves were used to compare 30-day survival across different risk levels.

**Results:**

Among 150 AAAD patients, NLL was recognized as a risk factor for 30-day mortality (hazard ratio = 1.83, 95% confidence interval: 1.29–2.58; *P* <0.001). The RCS analysis showed a linear relationship. NLL showed areas under the curve of 0.781, 0.781, and 0.730 for predicting 7-, 14-, and 30-day mortality, respectively. K–M curves revealed a significant difference in 30-day survival between the high- and low-risk groups (log-rank *P* = 0.042).

**Conclusion:**

NLL is a risk factor for 30-day mortality in AAAD patients and shows good predictive value. This study supports NLL as an early-warning biomarker for identifying high-risk AAAD patients.

## Introduction

Aortic dissection (AD) is a life-threatening cardiovascular disease (CVD) caused by a tear of the intima that separates the aortic wall into true and false lumens.^1,2)^ According to the Stanford classification system, dissections involving the ascending aorta are classified as type A, while those limited to the descending aorta are designated as type B.^3)^ Acute type A aortic dissection (AAAD) has pathological complexity and can directly involve the coronary artery opening, aortic valve, or pericardial cavity, causing acute myocardial infarction, aortic valve regurgitation, or pericardial tamponade, leading to cardiogenic shock.^4–6)^ The in-hospital mortality rate of AAAD is 22%, and most deaths occur within the first week, which is higher than the overall mortality rate of hospitalized patients with type B AD (13%).^7)^ It is worth noting that AAAD patients need to undergo emergency thoracotomy within the golden time window (<48 hours) to alleviate acute threats, but even if the surgery is successful, the postoperative mortality rate remains high.^8,9)^ In addition, despite advances in surgical techniques, improvements in the prognosis of AAAD patients still face challenges due to the complexity of the disease itself and the lack of effective risk models.^4)^ Therefore, there is an urgent need to identify biomarkers that can quickly evaluate the prognosis of AAAD patients, identify high-risk individuals, and guide personalized interventions.

Lactate is a product of anaerobic metabolism and is often used to assess the severity of tissue hypoperfusion and cellular hypoxia.^10)^ Lactate serves as a prognostic indicator for multiple CVDs. Fuernau et al.^11)^ discovered that the critical value of arterial lactate after 8 hours has the best discriminative power for the early prognosis of cardiogenic shock and can serve as a new treatment target. Research by Liang et al.^12)^ demonstrated a strong positive association between admission levels of lactate and 30- and 180-day mortality rates in patients with acute coronary syndrome. Elevated levels of lactate were identified as independent predictors of overall mortality at 30 and 180 days. Lactate concentration is a static indicator, reflecting only the balance between its production and clearance at a given time, whereas the dynamic indices can better reflect the steady-state changes of lactate.^13)^ The normalized lactate load (NLL), first proposed by Zhang et al.,^14)^ can dynamically evaluate the effect of lactate accumulation. Its calculation is based on the area under the curve (AUC) of the lactate–time curve divided by the total time, reflecting the average lactate load intensity per unit time. Compared with traditional static lactate measurement, the advantage of NLL lies in its ability to capture the fluctuation trend of lactate over time, rather than the instantaneous equilibrium state.^15,16)^ In addition, NLL can quantify the sustained exposure level of hyperprolactinemia, which may be beneficial for improving sensitivity to the severity of tissue hypoxia.^17,18)^

However, the predictive value of NLL for AAAD patients has not yet been elucidated. Therefore, this research applied NLL in the prognostic evaluation of AAAD and examined the relationship between NLL and the 30-day mortality rate in AAAD patients using data from the Medical Information Mart for Intensive Care-IV (MIMIC-IV) database, aiming to validate the potential prognostic value of NLL as a biomarker for AAAD patients.

## Materials and Methods

### Data source

This study utilized data extracted from MIMIC-IV (Version 2.2),^19)^ which is a comprehensive clinical database that includes clinical data of patients admitted to Beth Israel Deaconess Medical Center from 2008 to 2019. In this database, patient identifiers are removed for privacy reasons. The Institutional Review Board of the medical center has reviewed the MIMIC database and approved the informed consent and data-sharing initiatives. No additional ethical approval is required.

### Study population

**Fig. 1:**
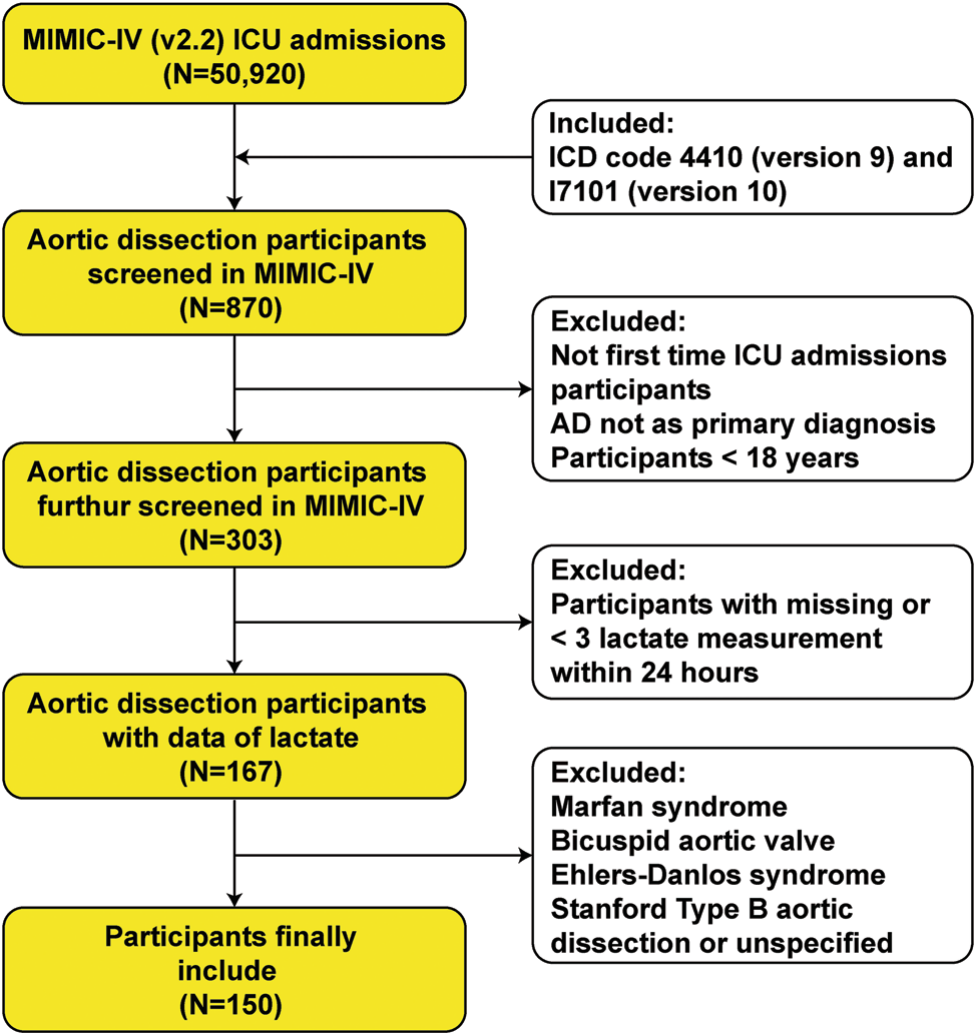
Flowchart of selecting participants. ICD: International Statistical Classification of Diseases; AD: aortic dissection; MIMIC-IV: Medical Information Mart for Intensive Care-IV; ICU: intensive care unit

This research identified 870 AAAD patients using diagnostic codes 4410 or I710 from the ninth (ICD-9) and tenth (ICD-10) revisions of the International Statistical Classification of Diseases (ICD).^20,21)^ Exclusion criteria included: (1) patients with multiple admissions, with AAAD as a non-primary diagnosis, or age under 18 years; (2) patients with less than 3 measurements of lactate within 24 hours of admission to the intensive care unit (ICU); and (3) patients with Ehlers–Danlos syndrome, Marfan syndrome, and congenital aortic valve abnormalities. A total of 158 samples were included, with 150 classified as Stanford A type and 8 classified as Stanford B type or undefined type. Therefore, 150 AAAD patients were included in this analysis (**Fig. 1**).

### NLL and mortality

We employed “lactate load” to indicate the AUC of lactate curves, which can explain the total effect of high lactate over time. “NLL” was used to indicate the AUC divided by time, which can explain the average intensity of high lactate.^13)^ The 30-day mortality rate in patients with AAAD was considered the primary outcome.

### Included variables

The variables collected in this study included demographic data (marital status, age, body mass index (BMI), race, height, gender, length of stay (LOS), weight), laboratory measurements (percutaneous oxygen saturation [SpO_2_], hematocrit, hemoglobin, glucose, prothrombin time [PT], NLL, chloride, bicarbonate, white blood cell [WBC] count, sodium, International Normalized Ratio (INR), lactate, platelets, mean corpuscular hemoglobin (MCH), partial thromboplastin time [PTT], potassium, anion gap, blood urea nitrogen [BUN], pH, MCH concentration, red blood cell [RBC], count, base excess, mean corpuscular volume [MCV], creatinine, red cell volume distribution width [RDW]), treatment methods (thoracic surgery), vital signs (mean blood pressure [MBP], respiratory rate, heart rate, systolic blood pressure [SBP], smoking status, diastolic blood pressure [DBP]), severity scores (Simplified Acute Physiology Score II [SAPS II], Logistic Organ Dysfunction Score [LODS], Glasgow Coma Score [GCS], Sequential Organ Failure Assessment [SOFA]), and medical history (myocardial infarction, liver disease, congestive heart failure, hypertension, diabetes, coronary artery disease). Laboratory measurement results, severity scores, vital signs, and other values measured multiple times during the ICU stay were recorded as the most severe values collected within the first 24 hours after admission to the ICU.

### Statistical analysis

We collected data from the MIMIC-IV (Version 2.2) database using Structured Query Language (SQL) and analyzed it with R software (Version 4.2.3; R Foundation for Statistical Computing, Vienna, Austria). Variables with missing values for vital characteristics and laboratory measurements accounting for less than 10% of the total samples were estimated using the random forest method in the “rice” package. Baseline tables were generated by using the “tableone” package. Continuous variables were expressed as mean ± SD. Comparisons between groups were performed using the *t*-test. The chi-squared test was applied to compare the categorical variables (expressed as percentages (%)), with a two-sided *P* value <0.05 indicating statistical significance.

The univariate and multivariate Cox proportional hazards models were employed to analyze the relationship between NLL and 30-day mortality in AAAD patients. Restricted cubic spline (RCS) curves were then drawn by using the “rms” package to illustrate the nonlinear relationship between NLL and the risk of death in AAAD patients. The “randomForestSRC” package was employed to establish a random survival forest model. The data of the samples were split into training and validation sets in a 5:5 ratio. Based on the training set, the receiver-operating characteristic (ROC) curves were plotted to evaluate the accuracy of the model. The maximum selection rank statistic method was applied using the “maxstat” package to determine cut-points that were closely associated with survival. Based on these cut-points, Kaplan–Meier (K–M) curves were drawn by using the “survival” package to compare the impact of 30-day survival status between high-risk patient groups.

## Results

### Baseline characteristics

We included 150 AAAD patients, of whom 20 (13.33%) died within 30 days of hospitalization. The patients were sorted into two groups: the survivor group (N = 130) and the non-survivor group (N = 20). **Table 1** displays the baseline characteristics of the two groups of patients. The average ages of the survivor group and non-survivor group were 64.40 ± 12.35 and 63.40 ± 17.63 years, respectively. The non-survivor group’s respiratory rate (19.47 ± 4.26 vs. 17.95 ± 2.82, *P* = 0.024) was significantly higher than that of the survivor group, whereas heart rate (77.21 ± 13.23 vs. 83.27 ± 11.14, *P* = 0.029) was significantly lower than that of the survivor group. Laboratory measurements demonstrated that NLL (4.44 ± 2.63 vs. 3.12 ± 1.58, *P* = 0.002) and lactate concentration (8.36 ± 3.90 vs. 5.50 ± 2.59, *P* <0.001) were significantly higher, while hemoglobin was significantly lower (7.44 ± 1.93 vs. 8.57 ± 1.87, *P* = 0.014). In terms of severity scores, the non-survivor group had significantly higher SAPS II, SOFA, and LODS scores than the survivor group (*P* <0.05). The analysis of medical history revealed no significant difference between the 2 groups (*P* >0.05).

**Table 1:** Baseline characteristics of patients

Characters	Total (N = 150)	Survivor (N = 130)	Non-survivors (N = 20)	*P*-Value
Age (years)	64.27 (13.11)	64.40 (12.35)	63.40 (17.63)	0.752
Gender				0.561
Female	55 (36.7)	46 (35.4)	9 (45.0)	
Male	95 (63.3)	84 (64.6)	11 (55.0)	
BMI (kg/m^2^)	29.66 (6.85)	29.23 (6.65)	32.32 (7.66)	0.068
Race				
White	73 (48.7)	67 (51.5)	6 (30.0)	0.136
Black	17 (11.3)	15 (11.5)	2 (10.0)	
Other race	60 (40.0)	48 (36.9)	12 (60.0)	
Marital status				0.377
Married	70 (46.7)	63 (48.5)	7 (35.0)	
Unmarried	80 (53.3)	67 (51.5)	13 (65.0)	
LOS (day)	9.77 (10.00)	9.65 (9.94)	10.51 (10.57)	0.722
Height (cm)	172.10 (10.89)	172.77 (10.63)	167.95 (11.87)	0.073
Weight (kg)	87.91 (22.42)	87.01 (21.36)	93.50 (28.18)	0.231
Smoke status	19 (12.7)	18 (13.8)	1 (5.0)	0.456
Heart rate (times/min)	82.47 (11.58)	83.27 (11.14)	77.21 (13.23)	0.029
SBP (mmHg)	111.23 (9.93)	111.10 (9.54)	112.09 (12.40)	0.681
DBP (mmHg)	58.95 (7.45)	58.88 (6.84)	59.40 (10.83)	0.773
MBP (mmHg)	74.86 (7.59)	74.94 (7.27)	74.32 (9.62)	0.735
Breath rate (times/min)	18.19 (3.09)	17.97 (2.81)	19.64 (4.31)	0.024
SpO_2_	97.22 (2.07)	97.21 (2.09)	97.35 (1.99)	0.778
Glucose (mg/dL)	140.43 (22.94)	139.76 (22.42)	144.79 (26.29)	0.364
SAPSII	43.29 (12.31)	42.24 (11.84)	50.15 (13.37)	0.007
SOFA	7.90 (3.51)	7.52 (3.38)	10.40 (3.44)	0.001
GCS	13.54 (3.57)	13.48 (3.65)	13.90 (3.01)	0.629
LODS	6.73 (2.80)	6.45 (2.71)	8.60 (2.66)	0.001
Normalized lactate load (mmol/L)	3.30 (1.80)	3.12 (1.58)	4.44 (2.63)	0.002
Chloride (mmol/L)	109.93 (4.05)	110.03 (4.01)	109.30 (4.34)	0.454
Hematocrit (μmol/L)	25.02 (5.54)	25.33 (5.52)	22.98 (5.37)	0.078
Hemoglobin (g/dL)	8.42 (1.91)	8.57 (1.87)	7.44 (1.93)	0.014
Potassium (K/μL)	4.66 (0.68)	4.61 (0.54)	4.93 (1.25)	0.053
Anion gap (mEq/L)	16.30 (4.93)	15.52 (3.90)	21.30 (7.47)	<0.001
Bicarbonate (mEq/L)	21.30 (3.32)	21.71 (2.95)	18.70 (4.34)	<0.001
Sodium (mEq/L)	139.25 (3.52)	139.16 (3.36)	139.80 (4.53)	0.455
PT (s)	19.45 (12.06)	19.21 (12.56)	21.00 (8.19)	0.539
PTT (s)	60.20 (37.63)	59.18 (37.18)	66.73 (40.77)	0.406
INR	1.71 (0.55)	1.68 (0.51)	1.93 (0.77)	0.058
Lactate (mmol/L)	5.89 (2.96)	5.50 (2.59)	8.36 (3.90)	<0.001
Platelets (K/μL)	115.79 (64.91)	118.82 (64.74)	96.20 (64.17)	0.148
WBC (K/μL)	14.94 (5.20)	14.81 (5.19)	15.78 (5.33)	0.441
RBC (M/μL)	2.79 (0.67)	2.83 (0.67)	2.52 (0.66)	0.06
pO_2_ (mmHg)	74.14 (28.26)	76.76 (27.82)	57.65 (25.89)	0.005
pCO_2_ (mmHg)	57.84 (15.93)	56.87 (15.49)	63.95 (17.65)	0.064
pH	7.22 (0.11)	7.23 (0.10)	7.13 (0.10)	<0.001
Base excess (mEq/L)	−6.85 (4.73)	−6.12 (4.33)	−11.45 (4.61)	<0.001
MCH (pg)	29.68 (1.97)	29.84 (1.89)	28.69 (2.23)	0.015
MCHC (g/dL)	32.69 (1.59)	32.89 (1.47)	31.34 (1.71)	<0.001
MCV (fL)	88.05 (4.96)	88.19 (5.13)	87.15 (3.65)	0.386
RDW	15.02 (1.72)	14.95 (1.77)	15.47 (1.38)	0.205
Open chest surgery	122 (81.3)	107 (82.3)	15 (75.0)	0.636
BUN (mg/dL)	24.11 (13.75)	22.31 (9.86)	35.70 (25.57)	<0.001
Creatinine (mg/dL)	1.74 (1.94)	1.52 (1.42)	3.13 (3.64)	<0.001
Myocardial infarct	6 (4.0)	6 (4.6)	0 (0.0)	0.713
Diabetes	17 (11.3)	14 (10.8)	3 (15.0)	0.860
Hypertension	122 (81.3)	109 (83.8)	13 (65.0)	0.088
Coronary artery disease	21 (14.0)	18 (13.8)	3 (15.0)	1.000
Congestive heart failure	15 (10.0)	14 (10.8)	1 (5.0)	0.689
Liver disease	9 (6.0)	6 (4.6)	3 (15.0)	0.189

BMI: body mass index; LOS: length of stay; SBP: systolic blood pressure; DBP: diastolic blood pressure; MBP: mean blood pressure; SpO_2_: percutaneous oxygen saturation; SAPSII: Simplified Acute Physiology Score II; SOFA: Sequential Organ Failure Assessment; GCS: Glasgow Coma Score; LODS: Logistic Organ Dysfunction Score; PT: prothrombin time; PTT: partial thromboplastin time; INR: International Normalized Ratio; WBC: white blood cell; RBC: red blood cell; MCH: mean corpuscular hemoglobin; MCHC: MCH concentration; MCV: mean corpuscular volume; RDW: red cell volume distribution width; BUN: blood urea nitrogen

### Association of NLL and lactate level on admission with 30-day mortality in patients with AAAD

To determine the impact of NLL and lactate levels on admission (maximum/minimum lactate levels on the first day of ICU) on the 30-day mortality rate of AAAD patients, a Cox regression model was created. In the unadjusted model, a significant association was observed between elevated NLL levels and increased 30-day mortality in AAAD patients (hazard ratio [HR] = 1.39, 95% confidence interval [CI]: 1.15–1.67; *P* <0.001) (**Table 2**). NLL was a risk factor for 30-day mortality in AAAD patients (HR = 1.83, 95% CI: 1.29–2.58; *P* <0.001) in the fully adjusted model. In addition, although the maximum lactate level on the first day was significantly correlated with the risk of mortality in the fully adjusted model (HR = 1.66, 95% CI: 1.32–2.08; *P* <0.001), its predictive power was still lower than that of NLL. The minimum lactate level on the first day had no predictive value (HR = 1.76, 95% CI: 0.98–3.15; *P* = 0.057).

**Table 2:** Multivariable Cox regression models evaluating the association between normalized lactate load, maximum and minimum lactate values, and hazard ratios (95% confidence intervals) for 30-day mortality

Outcomes	Normalized lactate load	Lactate max	Lactate min
Crude	1.39 (1.15–1.67), <0.001	1.36 (1.19–1.56), <0.001	1.30 (0.83–2.02), 0.250
Adjusted model	1.83 (1.29–2.58), <0.001	1.66 (1.32–2.08), <0.001	1.76 (0.98–3.15), 0.057

Crude was unadjusted. Adjusted model: Adjusted for age, gender, BMI, smoking status, heart rate, breath rate, white blood cell count, platelets, hemoglobin, potassium, BUN, diabetes, hypertension, LODS, GCS, SIRS and open chest surgery. Lactate measurements were collected within the first 24 hours of ICU admission.

min: minimum; max: maximum; BMI: body mass index; BUN: blood urea nitrogen; LODS: Logistic Organ Dysfunction Score; GCS: Glasgow Coma Score; SIRS, systemic inflammatory response syndrome; ICU: intensive care unit

**Fig. 2:**
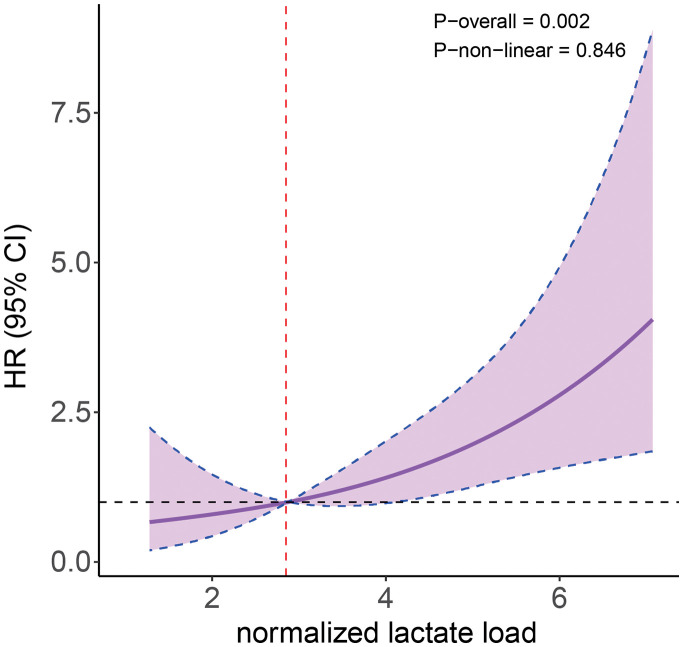
Association between NLL and 30-day mortality of participants. The Cox regression model with a restricted cubic spline shows a linear relationship between NLL and the risk of 30-day mortality in the patients. The solid line and shaded area represent the estimated values and their corresponding 95%. CIs, respectively. NLL: normalized lactate load, HR: hazard ratio; CI: confidence interval

A further RCS model was established to analyze the nonlinear relationship between NLL and 30-day mortality risk in patients with AAAD. After adjusting for all confounding factors, a significant association was observed between NLL and the 30-day risk of death in AAAD patients (*P* for overall = 0.002), which emerged as a significantly linear relationship (*P* for nonlinear = 0.846) with a threshold value of 2.87 (**Fig. 2**).

### The predictive value of NLL

**Fig. 3:**
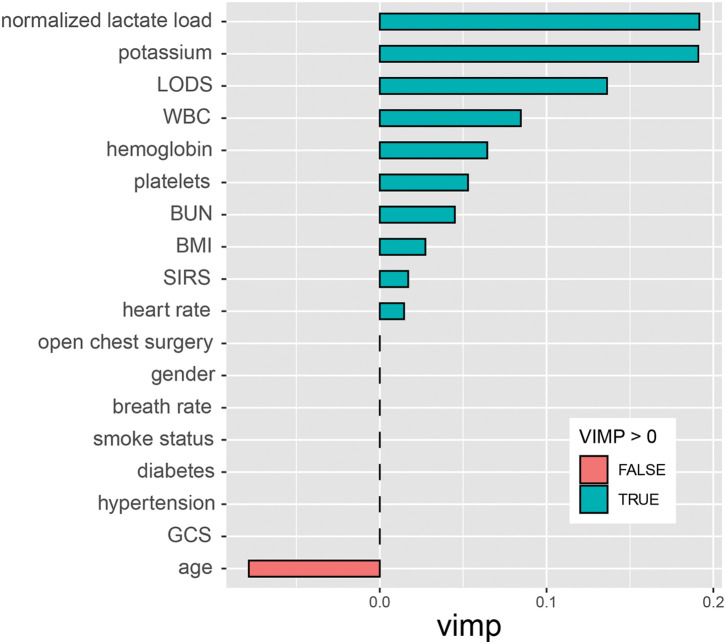
Variable importance of random survival forest. LODS: Logistic Organ Dysfunction Score; WBC: white blood cell; BUN: blood urea nitrogen; BMI: body mass index; SIRS, systemic inflammatory response syndrome; GCS: Glasgow Coma Score; VIMP, variable importance

**Fig. 4:**
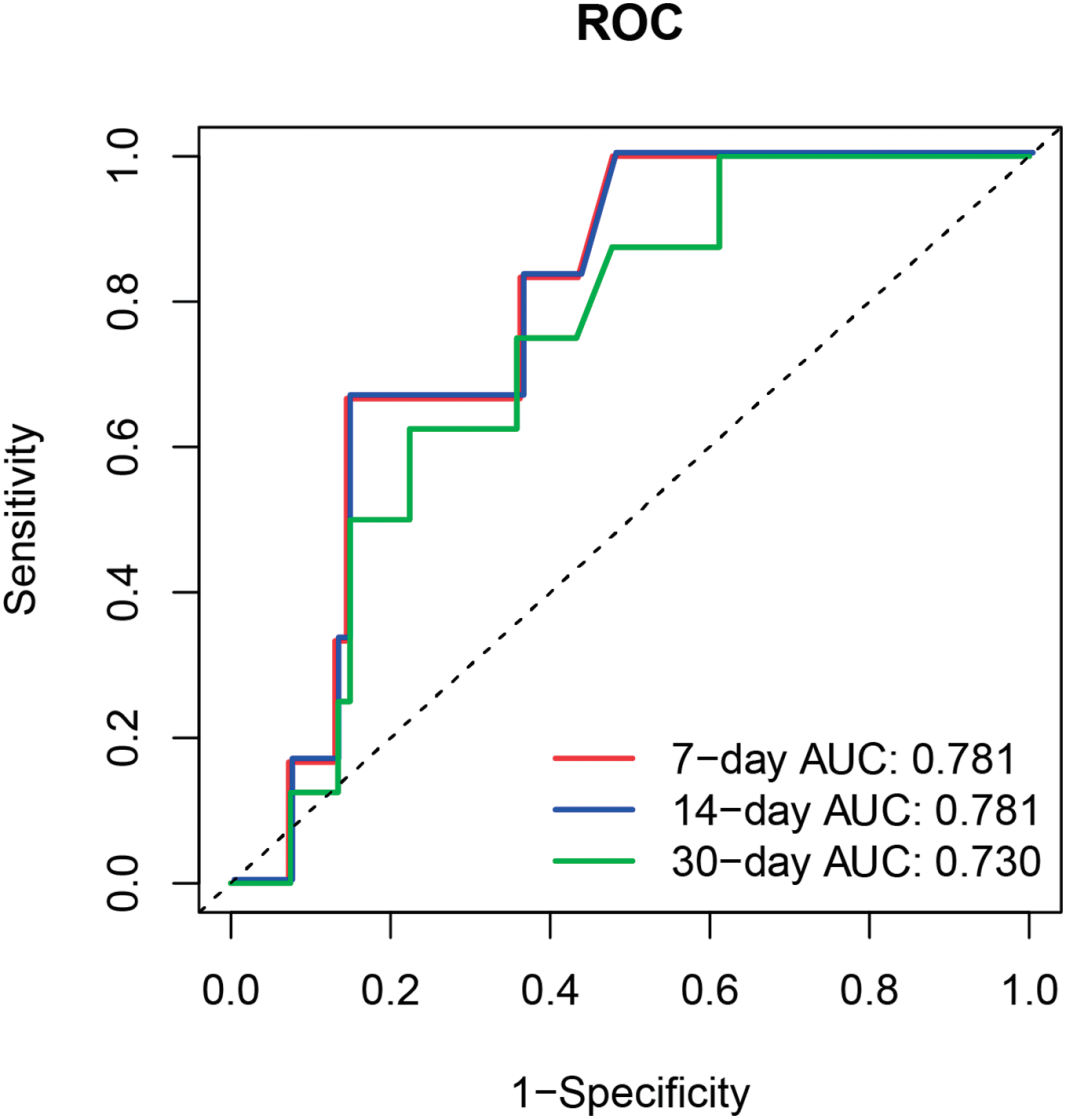
Time-dependent ROC curve of the random survival forest for testing data. ROC: receiver-operating curve; AUC: area under the curve

The predictive value of NLL was evaluated using a random survival forest prediction model and ROC curves. The top 5 variables that significantly influenced the model were NLL, blood potassium concentration, LODS, WBC, and hemoglobin (*P* <0.05) (**Fig. 3**). The ROC curves indicated that NLL predicted the 7-, 14-, and 30-day mortality rates of AAAD patients with AUC values of 0.781, 0.781, and 0.730, respectively (**Fig. 4**), with strong predictive value.

**Fig. 5:**
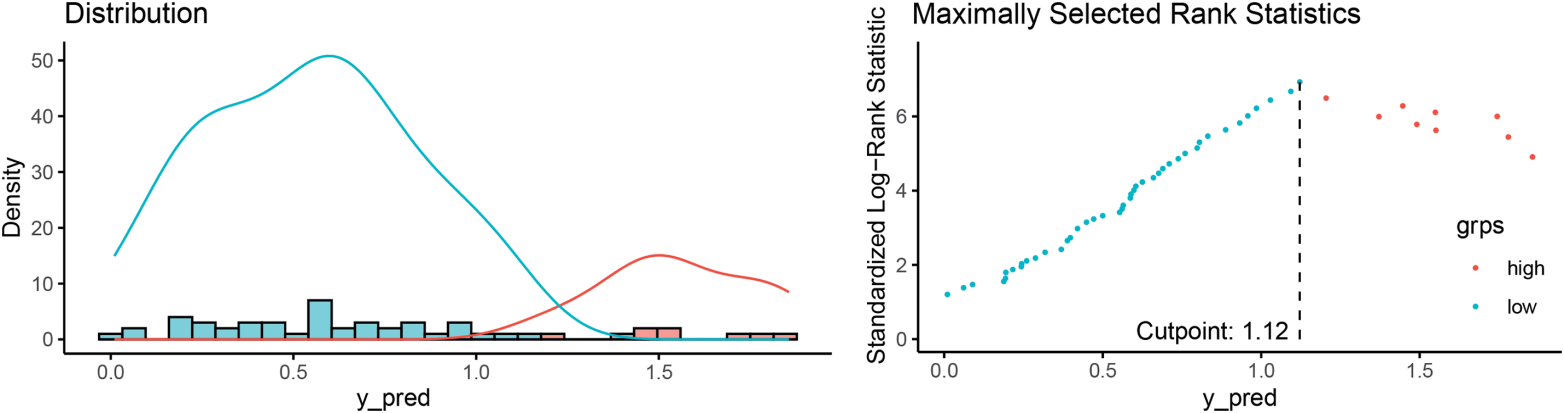
Cutoff risk points calculated by the maximally selected rank statistics. grps: groups

**Fig. 6:**
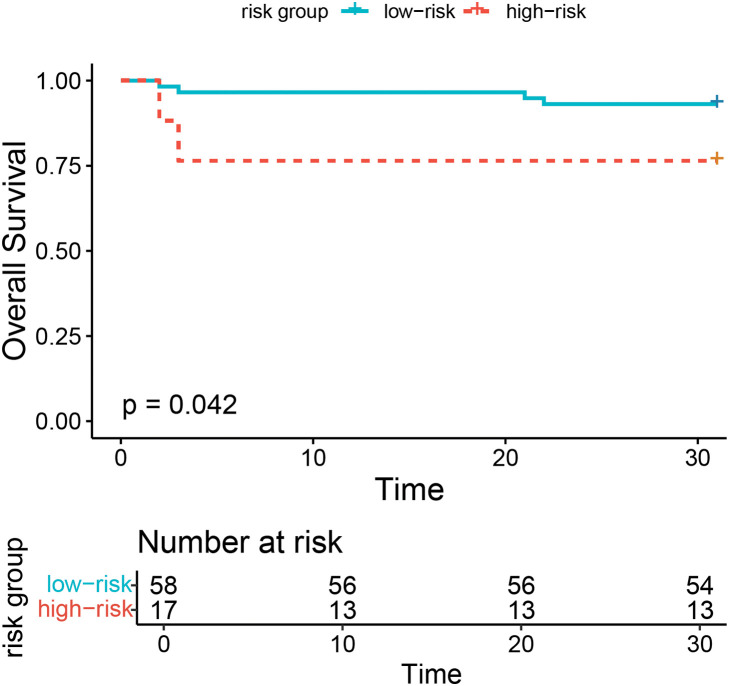
Survival curves showing the high- and low-risk groups in the testing datasets.

Based on a random survival forest model, risk scores were calculated to assess patient prognosis. The results indicated that the higher risk scores were associated with poorer prognoses. The maximum selection rank statistic from the “maxstat” package was applied to determine a cutoff risk score of 1.12 in the training set (**Fig. 5**). Based on the cutoff value, patients were clustered into high- and low-risk groups. K–M curves were plotted for the 2 groups. The log-rank test revealed that the survival rate of high-risk patients was significantly lower than that of the low-risk group (log-rank *P* = 0.042) (**Fig. 6**). The risk score clearly distinguished the patients with adverse prognoses.

## Discussion

Our findings identified NLL as a risk factor for 30-day mortality in patients with AAAD. The RCS analysis demonstrated a positive linear relationship. By comparing the ROC curves and AUC values, we found that NLL had a strong predictive value for the 7-, 14-, and 30-day mortality rates of AAAD patients.

Lactic acid, as a biomarker of tissue hypoperfusion, has been widely recognized to be closely associated with mortality in critically ill patients.^22,23)^ Hyperlipidemia is an independent predictor of increased mortality in the ICU populations.^24)^ The lactate level within 24 hours after admission to the ICU also has significant prognostic value.^25)^ It is worth noting that static lactate measurements reflect the metabolic status at only a single time point, whereas NLL dynamically quantifies the cumulative exposure of hyperprolactinemia by integrating the area under the lactate–time curve, which can reflect the duration of tissue hypoxia.^26)^ This advantage has been validated in multiple types of cardiovascular emergencies. NLL is an independent risk factor for mortality in patients with septic shock, and its accuracy is better than that of initial and maximum lactate values.^13)^ In predicting in-hospital mortality among patients with acute myocardial infarction, NLL outperforms both maximal and mean lactate values.^27)^ Moreover, in studies of patients with cardiogenic shock, the AUC value of NLL was significantly higher than that of other lactate indicators.^28)^ In the field of AAAD, although previous studies have suggested a correlation between high lactate levels and poor prognosis, these conclusions were based on static, single-point measurements that fail to capture the impact of dynamic lactate changes on prognosis. As reported in a relevant study, preoperative elevated lactate levels have been associated with increased mortality rates in postoperative patients,^29)^ and lactate levels within 6 hours of ICU admission have been shown to predict in-hospital mortality.^30)^ In this study, we introduced NLL and further confirmed that NLL is an independent risk factor for 30-day mortality in AAAD patients. In addition, the relationship between the first-day lactate extremum and prognosis in this study further confirmed the superiority of NLL as a dynamic indicator. Although the first-day maximum lactate level was significantly correlated with mortality risk (HR = 1.66, 95% CI: 1.32–2.08), its predictive power was still lower than that of NLL (HR =1.83, 95% CI: 1.29–2.58). The above results highlight the advantages of NLL as a dynamic indicator compared to static measurements, providing a more reliable metabolic basis for prognostic evaluation of AAAD.

The random survival forest model confirmed that NLL, blood potassium concentration, LODS, WBC, and hemoglobin constitute the core variable group for predicting the risk of death in AAAD patients, reflecting multiple pathophysiological processes. Blood potassium is an important blood parameter commonly applied in clinical practice and is closely associated with CVDs.^31,32)^ Admission serum potassium levels show a U-shaped relationship with both in-hospital and long-term mortality in AAAD patients, with blood potassium imbalance (levels outside the range of 3.5–4.5 mmol/L) serving as a risk factor for increased mortality.^33)^ This may be related to elevated serum potassium levels, which can induce arrhythmias and exacerbate hemodynamic disorders through myocardial inhibition.^34)^ LODS aims to integrate the severity measurement values of multiple organ dysfunction into a single score. An increase in the LODS score directly quantifies the degree of multi-organ failure, including the heart, liver, and kidneys,^35)^ which may form a vicious cycle with AAAD-related perfusion disorders through mechanisms such as endothelial dysfunction, lactate metabolism imbalance, and multi-organ failure.^36,37)^ Elevated WBC levels indicate activation of systemic inflammation. In AAAD, inflammatory mediators such as interleukin-6 and tumor necrosis factor-α are released by macrophages and neutrophils, promoting extracellular matrix remodeling of vascular walls, endothelial dysfunction, and false lumen formation. These processes further lead to thrombosis and reduced microcirculatory perfusion.^38–40)^ Low hemoglobin levels limit oxygen delivery capacity, serving as an independent predictor of postoperative mortality in AAAD patients.^41)^ Iron deficiency promotes aortic media degeneration, which is the core pathological basis of AAAD. Abnormal iron metabolism is involved in the development of dissection by regulating gene expression in vascular smooth muscle cells.^42,43)^ Low hemoglobin may reflect insufficient iron reserves, indirectly exacerbating aortic wall fragility, but this mechanism needs further validation. These variables may have an inherent synergistic mechanism with NLL, forming a core driver of the pathological network associated with high NLL and poor prognosis in AAAD.

Our research also revealed that NLL had strong predictive value for the 7-, 14-, and 30-day mortality rates of AAAD patients, which was in line with previous studies. In a retrospective study of patients undergoing AAAD surgery, the average lactate concentration in non-survivors was higher than in survivors, and postoperative lactate levels demonstrated moderate predictive ability for in-hospital mortality.^30)^ According to a study on the preoperative predictive indicators, the levels of serum lactate levels were positively associated with the one-year mortality rate, indicating that the level of lactate is a preoperative predictive factor for the one-year mortality rate in AAAD patients.^44)^ An analysis of prognosis demonstrated that postoperative lactate is closely associated with in-hospital mortality in patients undergoing surgery, suggesting that lactate could serve as a potential predictor of in-hospital mortality.^45)^ The formation of AD is mainly due to the entry of blood from the aortic lumen into the media of the aortic wall through a tear in the aortic intima, causing separation of the media and resulting in the formation of true and false lumens within the aortic wall.^46)^ The extended false lumen can obstruct blood flow in the true lumen, leading to poor perfusion of the coronary arteries, brain, and visceral blood vessels.^47)^ When oxygen delivery and tissue perfusion decline, the levels of lactate rise.^48)^ Under hypoxic conditions, pyruvate cannot enter the mitochondria for aerobic oxidation, and tissue cells shift to anaerobic metabolism to produce energy. The main product of anaerobic metabolism is lactate.^49)^ In addition, complications of AD, such as renal failure, can cause organ failure, which can lead to reduced lactate clearance.^50–52)^ This may further cause the accumulation of lactate.

The prognosis of AAAD patients is also significantly influenced by anatomical features and surgery-related factors, which may have potential interactions with NLL. From an anatomical perspective, the extent of dissection, perfusion status of the false lumen, and degree of aortic valve regurgitation directly determine the blood flow perfusion of important organs, which in turn affects the occurrence and duration of tissue hypoxia and affects NLL levels by altering lactate production and clearance dynamics.^53–55)^ Surgery-related factors are equally critical, including the timing of emergency surgery (whether performed within the golden time window <48 hours), surgical approach, the occurrence of complications such as aortic rupture or pericardial tamponade during surgery, and postoperative false lumen closure. All these factors may affect the dynamic changes in lactate metabolism by altering blood flow stability and tissue perfusion, thereby contributing to prognosis regulation together with NLL.^56,57)^ Therefore, when evaluating the predictive value of NLL in clinical practice, it is necessary to combine these anatomical and surgical variables for comprehensive analysis. NLL, as a metabolic indicator reflecting dynamic changes in tissue hypoxia, can complement the above factors and jointly improve the comprehensiveness and accuracy of prognostic evaluation for AAAD patients.

In this study, NLL, as an important prognostic indicator for AAAD patients, has significant clinical value. There is a significant difference in the mechanism of lactate elevation between high lactate levels in AAAD patients and those in other types of shock, such as septic shock and cardiogenic shock. The latter are often caused by systemic inflammation or myocardial pump failure,^15,58)^ while the core mechanism of elevated lactate in AAAD lies in regional perfusion disorders caused by aortic anatomical structural damage.^59,60)^ Therefore, relieving anatomical obstruction is an important measure to improve lactate levels. It must be emphasized that emergency surgical repair is the only effective treatment method for AAAD, and this decision does not change regardless of the level of NLL. For high-risk AAAD patients with NLL >2.87, the clinical significance of NLL lies in identifying individuals with an extremely high risk of death to warn the clinical team to make more adequate preoperative preparations (such as optimizing mechanical circulatory support, and blood product preparation) and implement more proactive perioperative management for such patients to cope with possible multiple organ failure. This NLL-based risk stratification provides a key perspective for cardiovascular surgeons to distinguish the mechanisms of elevated lactate levels in AAAD from those in other shock states, which can help optimize perioperative support strategies more accurately in emergency surgery, potentially improving patient prognosis.

Nevertheless, this study has certain limitations. First, as a retrospective study, bias may inevitably influence the reliability of the results. The causal link cannot be determined, necessitating further validation in larger populations. Second, the specific etiology of each AAAD case was difficult to determine. Diagnosis based solely on ICD codes rather than standardized clinical criteria may have influenced the accuracy of AAAD cases. Third, given the limitations of public databases, whether treatment measures can reduce NLL is still undetermined. Therefore, whether reducing NLL improves the long-term prognosis of patients remains elusive. Fourth, as MIMIC-IV did not record surgical details such as CPB time and aortic repair range, these potential confounding factors may affect the interpretation of the association between NLL and prognosis, and further prospective studies are needed to verify this. Fifth, the conclusion of this study is applicable only to patients with AAAD. In the future, the B-type AD cohort needs to be expanded to validate the subtype-specific predictive value of NLL. Finally, this study included only cases with 3 or more lactate measurements within 24 hours of admission to the ICU, which may have excluded some critically ill AAAD patients requiring emergency surgery and introduced selection bias. Future research should aim to include a more comprehensive cohort, including patients undergoing emergency surgery, and explore more flexible lactate monitoring strategies to validate the generalizability of the results of this study.

## Conclusion

The results of this study indicated that NLL is an independent risk factor for the 30-day mortality rate in AAAD patients and has good predictive value for the 7-, 14-, and 30-day mortality rates of AAAD patients. Its dynamic evaluation advantage is significantly better than that of static lactate measurement and it can provide reliable metabolic evidence for the early identification of high-risk patients. This finding supports NLL as a potential biomarker for the prognostic evaluation of AAAD, which is beneficial for assisting cardiovascular surgeons in distinguishing the mechanism of lactate elevation between AAAD and other shock states and provides important references for optimizing emergency surgical timing and perioperative management strategies. Nevertheless, further research is required to determine whether reducing NLL can improve clinical outcomes in AAAD patients.

## Declarations

### Data availability

Data sharing is not applicable to this article as no datasets were generated or analyzed during the current study.
